# Human donor milk for the vulnerable infant: a Canadian perspective

**DOI:** 10.1186/1746-4358-9-4

**Published:** 2014-04-17

**Authors:** Julia Panczuk, Sharon Unger, Deborah O’Connor, Shoo K Lee

**Affiliations:** 1 Department of Medicine, University of Toronto, Toronto, ON, Canada; 2 Division of Neonatology, The Hospital for Sick Children, 555 University Avenue, Toronto, ON M5G 1X8, Canada; 3 Department of Paediatrics, University of Toronto, Toronto, ON, Canada; 4 Department of Paediatrics, Mount Sinai Hospital, 600 University Avenue, Toronto, ON M5G 1X5, Canada; 5 Department of Nutritional Sciences, University of Toronto, Fitzgerald Building, 150 College Street, Toronto, ON M5S 3E2, Canada; 6 Research Institute, The Hospital for Sick Children, Toronto, Canada; 7 Department of Obstetrics and Gynaecology, University of Toronto, Toronto, ON, Canada

**Keywords:** Breast milk, Mother’s own milk, Pasteurized human donor milk, Very low birth weight infants

## Abstract

Breast milk is the normal way to feed infants and is accepted worldwide as the optimal first source of nutrition. Though the majority intend to breastfeed, many mothers of sick, hospitalized newborns, particularly those of very low birth weight, are unable to provide a full volume of milk due to numerous physical and emotional barriers to breastfeeding. This vulnerable population of infants may benefit most from receiving breast milk nutrition and thus pasteurized donor milk should be the first consideration for supplementation when there is an inadequate supply of mother’s own milk. This commentary will briefly review the history of milk banking in Canada, as well as the best available evidence for donor milk use in the very low birth weight population, including available economic analyses, with a view to advocate for its use in these vulnerable infants.

## Background

Breast milk is the normal way to feed infants and is accepted worldwide as the optimal exclusive first source of nutrition [1-5]. The overwhelming majority of Canadian mothers wish to breastfeed. For mothers of vulnerable infants, however, particularly those of very low birth weight (VLBW, <1500 g) or requiring gastrointestinal surgery, it is more than simply a matter of desire. Despite their best intentions, only a minority can produce a full volume of milk due to numerous physical and emotional barriers to breastfeeding, including maternal illness, stress, immature lactocytes, and separation from their infant. In our neonatal intensive care unit (NICU), the largest in Canada, only 30% of mothers are able to achieve exclusive breastfeeding. Until very recently, preterm formula was offered in most Canadian NICUs as a standard supplement in the absence of mother’s own milk. It is in this vulnerable population of infants, however, that the benefits of breast milk may be most important. Our first priority must be to support mothers in the provision of their own milk and our second priority should be to have donor milk routinely available as a safe, efficacious and cost-effective supplement when it is not possible to give mother’s own milk [6].

## Discussion

The Canadian Paediatric Society and the American Academy of Pediatrics have recommended fortified breast milk as first-line nutrition for preterm and other high-risk infants since the mid to late 1990s [7,8]. The benefits of breast milk compared with formula in VLBW infants are well established. Preterm infants fed breast milk display improved feeding tolerance [9], develop fewer severe infections [10,11] and fewer episodes of necrotizing enterocolitis (NEC) [12], are less colonized by pathogenic organisms [13,14], and experience decreased lengths of hospital stay [15] and reduced rates of hospital re-admission after discharge [16]. Importantly, breast milk-fed preterm infants also have improved neurodevelopmental outcomes. A meta-analysis by Anderson et al. showed a significant increase in measures of cognitive function among breastfed infants compared with their formula-fed counterparts [17]. The effect of breast milk was greatest for LBW infants, and held true after adjusting for possible socioeconomic confounders. A recent large-scale observational study by Vohr et al. assessing early nutrition of extremely LBW infants found a dose-dependent response to breast milk exposure on cognitive outcome as assessed by the Bayley Mental Developmental Index at 18 months of age [18]; this effect persisted at 30 months [16]. The mechanism for improved cognitive outcomes is likely multifactorial: breast milk promotes optimal feeding tolerance, provides an optimum substrate for brain and somatic growth, and provides protection against many complications associated with preterm birth that may negatively impact neurodevelopment [9-12,15].

**Figure 1:**
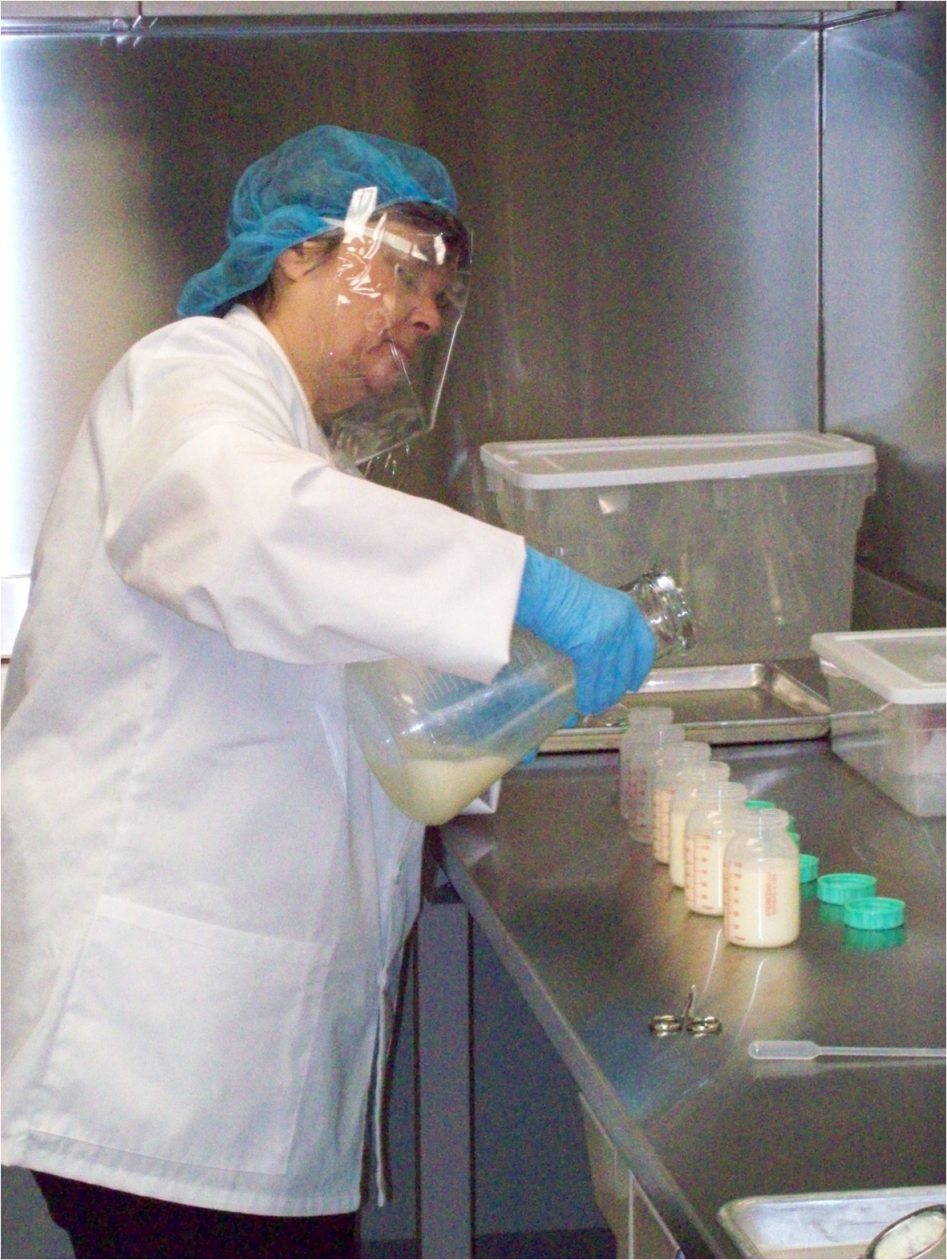
**Processing donor milk at the Rogers Hixon Ontario Human Milk Bank.** Donor milk is collected from across Ontario, Canada, processed, and redistributed through the Rogers Hixon Ontario Human Milk Bank. The milk bank dispensed 18,000 ounces of milk in the first 8 months since it opened.

The first human milk bank opened in Vienna, Austria in 1909 and the first in North America opened in 1919 in Boston, USA [19,20]. Banks continued to be established until the emergence of the HIV epidemic in the mid 1980s when many closed their doors in response to uncertainty of disease transmission via donor milk. With advances in donor screening and infectious disease testing assuring its safety, as well as mounting evidence for the benefit of breast milk in general, interest and demand has increased exponentially for donor milk. Currently there are 13 non-profit donor milk banks in North America. In addition to a long-standing bank in Vancouver, Canada now has a second operational bank in Calgary, a third in Ontario (see Figure 1), and one in development in Quebec [20]. In Canada, donor milk has been advocated for by the Canadian Paediatric Society [6]. It is regulated by Health Canada under food guidelines and all banks follow donor screening protocols established by the Canadian Blood Services. Donor milk has been widely accepted as shown by the example in Ontario, Canada’s largest province, where all tertiary care NICUs began importing donor milk within 6 months of the opening of the provincial milk bank. All Canadian banks are members of the Human Milk Banking Association of North America (HMBANA), whose policies guide the processing of human milk. The amount of milk dispensed by HMBANA banks rose from 409,077 ounces in 2000 to over 2 million ounces in 2011, a greater than 400% increase [20].

**Figure 2:**
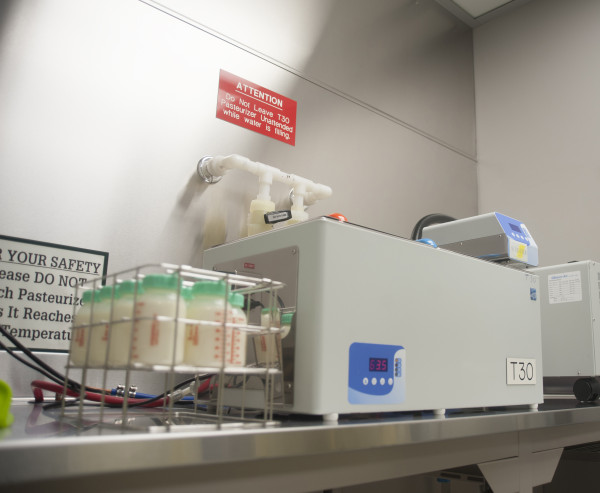
**Pasteurization of donor milk.** In North America, donor milk is pasteurized according to the Holder technique, which involves heating the milk to 62.5°C/144.5 °F and holding this for 30 minutes.

In North America, donor milk is pasteurized according to the Holder technique (see Figure 2) which, in combination with at least one additional container change and freeze/thaw cycle, can affect nutrient composition. This issue can be addressed by nutrient fortification of milk as required. However, pasteurization is known to have a greater impact on some of the bioactive components in milk [21]. For this reason, the efficacy of donor milk must be considered separately from mother’s own milk when reviewing the literature. The majority of donor milk research was generated prior to 1985 when many milk banks closed. The best evidence for its efficacy is a Cochrane review by Quigley et al. [22]. This meta-analysis included 8 randomized controlled trials that compared formula feeding versus human donor milk in preterm infants and found higher rates of diarrhea and feeding intolerance among formula-fed infants, and more importantly, significantly higher rates of NEC. The review did not show an effect on long-term growth or development, although those infants fed formula displayed higher short-term rates of growth. The latter finding is not unexpected, as only one trial used nutrient-fortified donor milk—a practice that is now standard [7,8]. All but one trial were also over 20 years old, conducted at a time when morbidity and mortality for VLBW infants were far greater and feeding practices did not reflect preferential use of breast milk.

It is reasonable to expect that if similar trials were conducted today, infants fed fortified donor milk would not only experience improved growth, but many of the other beneficial effects of breast milk as well. One recent industry-sponsored trial demonstrated that an exclusively human milk-based diet, including human milk-based human milk fortifier (HMF) and donor milk, resulted in a significant reduction in medical and surgical NEC in extremely preterm infants compared with those supplemented with bovine-based products [23]. At present, there are two multi-centre randomized control trials underway whose aim is to assess the impact of receiving donor breast milk as compared with preterm formula when mother’s milk is not available for very low birth weight infants: the American Milk Trial [24] and the Canadian Donor Milk for Improved Neurodevelopmental Outcomes Trial [25]. These are both large-scale trials, reflective of current era feeding practices in the most vulnerable infants, and have a primary outcome of long-term neurodevelopment as assessed by the Bayley Scales of Infant Development at 18 months corrected age.

There are also financial considerations in the use of donor breast milk. Operating a milk bank, including donor testing as well as processing, testing, and shipping donor milk entails a cost. However, in comparison with the cost of medical or surgical management of even one case of NEC or a resulting case of short bowel syndrome, this cost is nominal. Though there have been no Canadian analyses of the potential economic impact of donor milk, a Californian study estimated a cost savings to the healthcare system of $11 for every $1 spent on donor milk as a result of the reduction in hospital stay, NEC and sepsis associated with its use [26]. A recent study evaluating the cost-effectiveness of a 100% human milk-based diet including human milk-based fortifier for extremely preterm infants compared with bovine-based fortifier estimated a net savings of 3.9 NICU days and $8,167.17 per infant in preventable costs for NEC [27].

## Conclusion

Optimizing nutrition for preterm infants is of critical importance in promoting their health and improving their long term neurodevelopmental outcome. We must therefore advocate for improved supports enabling mothers to provide their own milk for their infant, as well as advocate for donor milk banking as the superior alternative in the absence of mother’s own milk.

## Abbreviations

HMBANA: Human Milk Banking Association of North America; HMF: Human milk fortifier; NEC: Necrotizing enterocolitis; NICU: Neonatal intensive care unit; (V)LBW: (Very) low birth weight.

## Competing interests

The authors declare that they have no competing interests. No specific sources of funding were used in the writing of this article.

## Authors’ contributions

SU, JP and DOC conducted the literature review, JP wrote the manuscript, and SKL provided senior authorship and draft revision. All authors read and revised the manuscript critically for important intellectual content and approved the final manuscript.

## Authors’ information

JP is Clinical Fellow Neonatology, Department of Medicine, University of Toronto. SU is Staff Neonatologist, Department of Paediatrics, Mount Sinai Hospital and Assistant Professor, Department of Paediatrics, University of Toronto. DO is Professor, Department of Nutritional Sciences, University of Toronto and Senior Associate Scientist, Research Institute, The Hospital for Sick Children. SKL is Paediatrician-in-Chief, Mount Sinai Hospital and Professor, Departments of Paediatrics and Obstetrics and Gynaecology, University of Toronto.
